# Development of an efficient root transgenic system for pigeon pea and its application to other important economically plants

**DOI:** 10.1111/pbi.13101

**Published:** 2019-03-27

**Authors:** Dong Meng, Qing Yang, Biying Dong, Zhihua Song, Lili Niu, Litao Wang, Hongyan Cao, Hanghang Li, Yujie Fu

**Affiliations:** ^1^ Beijing Advanced Innovation Center for Tree Breeding by Molecular Design Beijing Forestry University Beijing China; ^2^ The College of Forestry Beijing Forestry University Beijing China; ^3^ Key Laboratory of Forest Plant Ecology Ministry of Education Northeast Forestry University Harbin China

**Keywords:** *Agrobacterium rhizogenes*, hairy root, non‐model plants, transgenic root

## Abstract

For non‐model plants, functional characterization of genes is still hampered by lack of efficient stable transformation procedures. Here, we report a simple, fast and efficient transformation technique with *Agrobacterium rhizogenes* for generating stable transgenic roots in living plants to facilitate functional studies *in vivo*. We showed that injection of *A*. *rhizogenes* into stems of various plant species lead to stable transgenic root generation, which can sustain plant growth after the original, non‐transgenic roots were cut off. A transformation system was established for pigeon pea, a major woody food crop, after optimizing the selection of *A. rhizogenes* strains, bacterium concentration, injection position and seedling age. RT‐PCR and fluorescence observation indicated a transgenic root induction efficiency of about 39% in pigeon pea. Furthermore, induction of hairy roots was achieved in nine out of twelve tested economically important plants at an efficiency of 15–39%. As proof of concept, bimolecular fluorescence complementation (BiFC) assay was applied to test the interaction between CcCIPK14 and CcCBL1/2 in pigeon pea. Additionally, ectopic expression of the bZIP transcription factor MdHY5 from apple confirmed the utility of the transformation technique for engineering anthocyanin synthesis in roots. Taken together, we show that this method allows fast *in vivo* studies of gene function in a wide range of plant species.

## Introduction

*Agrobacterium*‐mediated plant transformation system has been the most widespread and successful method for plant genetic engineering in recent decades (Gelvin, 2003; Matveeva and Lutova, 2014; Vain, 2007). *Agrobacterium tumefaciens* invades plants at wounds where it can cause tumours by transferring a tumour‐inducing (Ti) plasmid to the plant cell nucleus. Based on this mechanism, the Ti plasmid was modified and tumour‐inducing genes were replaced by genes of interest to be incorporated into the chromosomes of the plant cell. Since the first widely accepted demonstration of successful transfer of exogenous genes using the *Agrobacterium*‐mediated plant transformation method in tobacco in 1983, efficient transformation systems have been established for a wide range of model plants and crops facilitating gene functional research and plant genetic modifications (Zhang *et al*., 2006; Wenzler *et al*., 1989; Ishida *et al*., 1996). However, unique medicinal or woody plant species with long growth cycles are still lacking efficient transformation and rapid screening systems, strongly hampering research on these species with genetic approaches (Clough and Bent, 1998; Hoekema *et al*., 1989; Mrízová *et al*., 2014).

*Agrobacterium rhizogenes* (*Rhizobium rhizogenes*) is a relative of *Agrobacterium tumefaciens* and can be used to induce adventitious roots named ‘hairy roots’ upon wounding and infection of plant leaves or stems (Ozyigit *et al*., 2013). A target gene can be transferred and incorporated into the genome of the host plant by infection of *R. rhizogenes* harbouring a modified Ri plasmid, resulting in transgenic hairy roots. Based on this mechanism, a valuable biotechnological application was exploited, known as hairy root culture (HRC). In the past decade, HRC was extensively applied to a wide variety of plant species (Shajahan *et al*., 2017; Veena and Taylor, 2007) for a diverse range of biotechnological studies, such as phytochemical and recombinant protein production, analyses of rhizosphere physiology and biochemistry and molecular breeding. For example, overexpression of the geraniol 10‐hydroxylase (G10H) gene or a jasmonate‐responsive APETALA2 (AP2)‐domain transcript factor in HRC, improves catharanthine production in *Catharanthus roseus* (Paul *et al*., 2017). Manipulations of structural genes/transcriptional factors, culture media and nutrition of the HRC system have allowed researchers to identify a remarkable range of main flavones (Dubos *et al*., 2010; Jiang *et al*., 2010; Yang *et al*., 2012, 2017; Zhang *et al*., 2009). The HRC system has also been utilized to uncover the function of PvPOX1 (plant peroxidases) in Fusarium wilt resistance of three common bean varieties (Estrada‐Navarrete *et al*., 2006, Xue *et al*., 2017). Although the hairy root system has proven to be very valuable to investigate gene functions, there are very few reports on medicinal and woody plants.

In this study, we developed a simple, fast and efficient root transformation system for a wide range of medicinal and woody plant species that allows functional characterization of genes. We first established an Agrobacterium‐mediated induction of hairy roots in pigeon pea, a woody food crop and identified Agrobacterium K599 having the highest induction rate. Then, we optimized the transformation procedure for high transgenic rate in pigeon pea and extended the application of this method to eleven other economically important plants including herbs, shrubs and trees. Finally, we demonstrated the potential of the hairy root transgenic system to facilitate research on medicinal and woody plants with bimolecular fluorescence complementation (BiFC) assay of protein‐protein interactions and engineering anthocyanin synthesis in roots.

## Results

### Transgenic hairy root induction in pigeon pea

**Figure 1:**
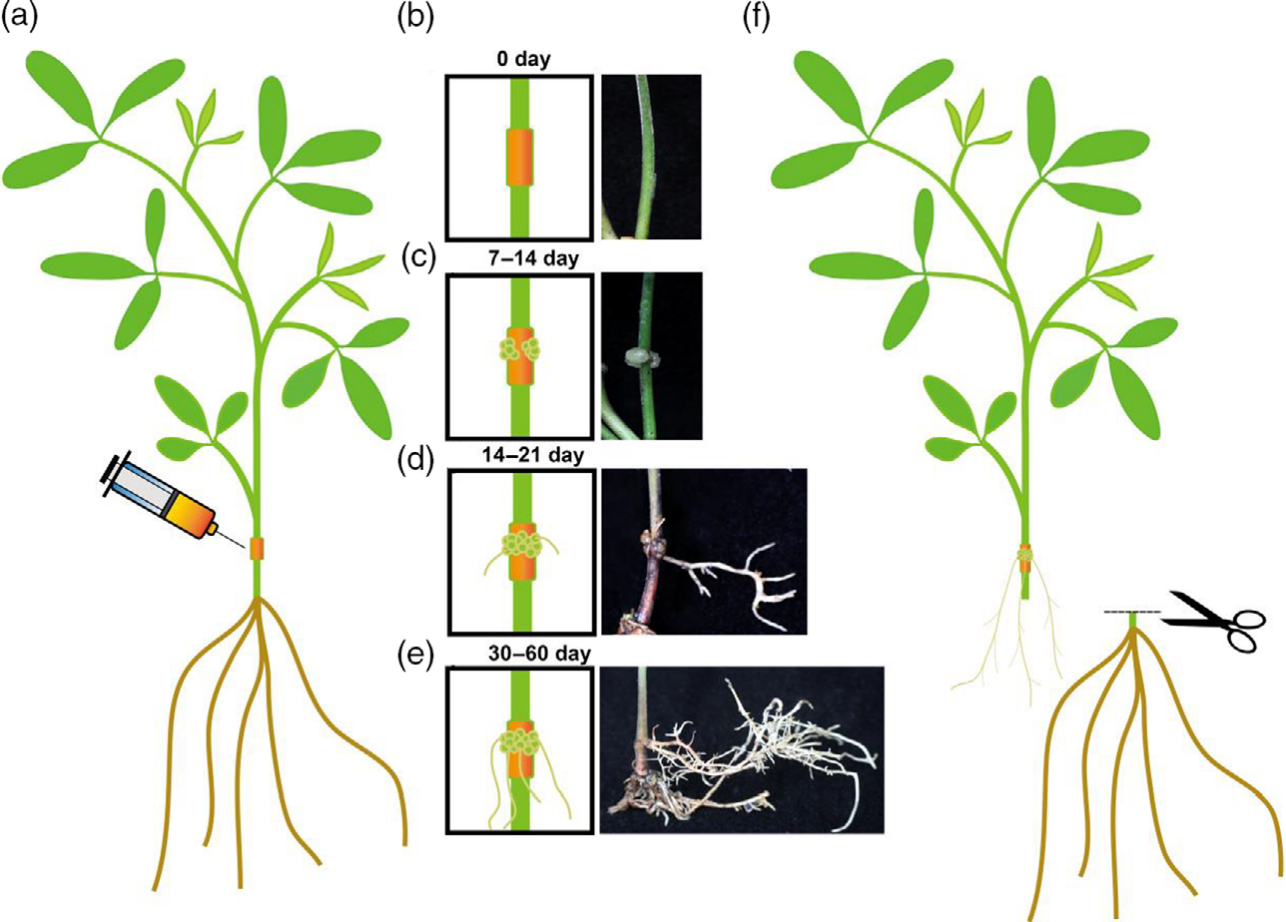
The hairy root transgenic system in pigeon pea. (a) The *Agrobacterium rhizogenes* solution carrying the vectors injected into the stem of one‐month old, sub‐cultured pigeon pea seeding. (b‐e) The process of callus and hairy root regeneration. Left is the schematic diagram for each stage; right is the picture corresponding to each stage. (f) The final step of cutting off the original roots after transgenic hairy roots fully developed.

In this study, we developed an efficient agrobacterium injection system in pigeon pea seedlings. As shown in figure 1, the constructed vector was introduced into the *Agrobacterium rhizogenes* strains and then injected into 15‐day‐old pigeon pea seedlings (Figure 1a). After about 7–14 days of growth, calli appeared around the injection site and gradually expanded (Figure 1b,c). After about three weeks, small hairy roots, similar to adventitious roots, grew from the calli (Figure 1d). After one to two months, the hairy roots developed so extensively that they could support the whole plant alone (Figure 1e). At this stage, we tested whether the target gene had been successfully introduced into those hairy roots through RT‐PCR or using western blot. After confirmation of the transgenic hairy roots, the original roots were cut off and the whole plant was solely supported by the transgenic hairy roots (Figure 1f). This provides a very fast and efficient system for gene functional analysis, secondary metabolites engineering and plant stress response studies. In this system, because only the roots were transgenic, it may also make it possible to study signal transduction between roots and shoots.

### Selection of agrobacterium strains, injection concentration and injection site

In order to select one *A. rhizogenes* strain that gives the highest transformation efficiency in pigeon pea, *A. rhizogenes* strains K599, MSU440, C58C1 and ArA4 were tested in pigeon pea seedlings. Different *Agrobacterium* solutions with GFP plasmids were injected into 15‐day‐old seedlings to induce hairy roots. As shown in Table S1, *A. rhizogenes* strain K599 exhibited the highest induction efficiency of calli (80%) and roots (30%) among all the strains tested. For calli, the induction efficiency of strain C58C1 was only 15%, followed by MSU440 (10%) and ArA4 (8%). For hairy roots, the induction efficiencies of these three strains were only 5–8%. So K599 is most suitable for inducing hairy roots in pigeon pea.

**Figure 2:**
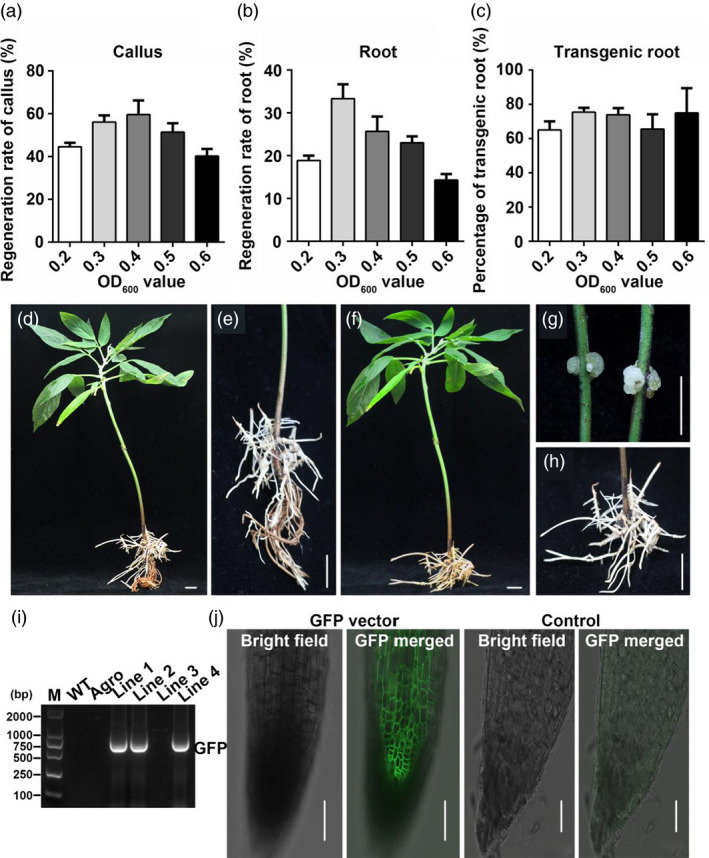
Selection of the optimal concentration of bacteria solution for infection and identification of transgenic lines. OD_600_ values of 0.2, 0.3, 0.4, 0.5 and 0.6 were tested. (a) The regeneration rate of callus after injecting different OD_600_ values of agrobacterium strain K599. (b) The regeneration rate of hairy roots in different OD_600_ values of agrobacteria solution. (c) Transgenic rate of regeneration hairy roots under different OD_600_ values. (d‐e) Transgenic hairy roots with its original, non‐transgenic roots. G Callus regeneration from stem after injection in pigeon pea. (f–h) Transgenic hairy roots after cutting off the original roots. Bars in D to H is 1 cm. (i) RT‐PCR analysis of the transgenic rate of hairy roots. (j) GFP signal in transgenic hairy roots, with empty vector‐containing agrobacterium solution as control. Bars in J is 50 μm.

Next, we tested a range of concentrations of *Agrobacterium* solution (0.2, 0.3, 0.4, 0.5 and 0.6 OD_600_ values) for callus induction rate, hairy root induction rate and transgenic rate. The results showed that OD_600_ values of 0.3 and 0.4 were optimal for getting a high callus induction rate (58 ± 2% and 60 ± 4% respectively) in pigeon pea seedlings (p < 0.05) (Figure 2a). The OD_600_ value of 0.3 also showed the best regeneration rate of hairy roots (33 ± 4%), followed by the OD_600_ value of 0.4 (26 ± 4%) (Figure 2b). Moreover, there were no differences of the transgenic root rates between the different *Agrobacterium* solution concentrations (Figure 2c). In summary, the optimal concentration for both callus and hairy root induction was OD_600_ 0.3. The phenotype of transgenic hairy roots before and after being cut and the original root of pigeon pea is shown in Figure 2d–h. The PCR results and GFP‐signals both confirmed that the target gene had been transferred into the hairy roots (Figure 2i,j).

**Figure 3:**
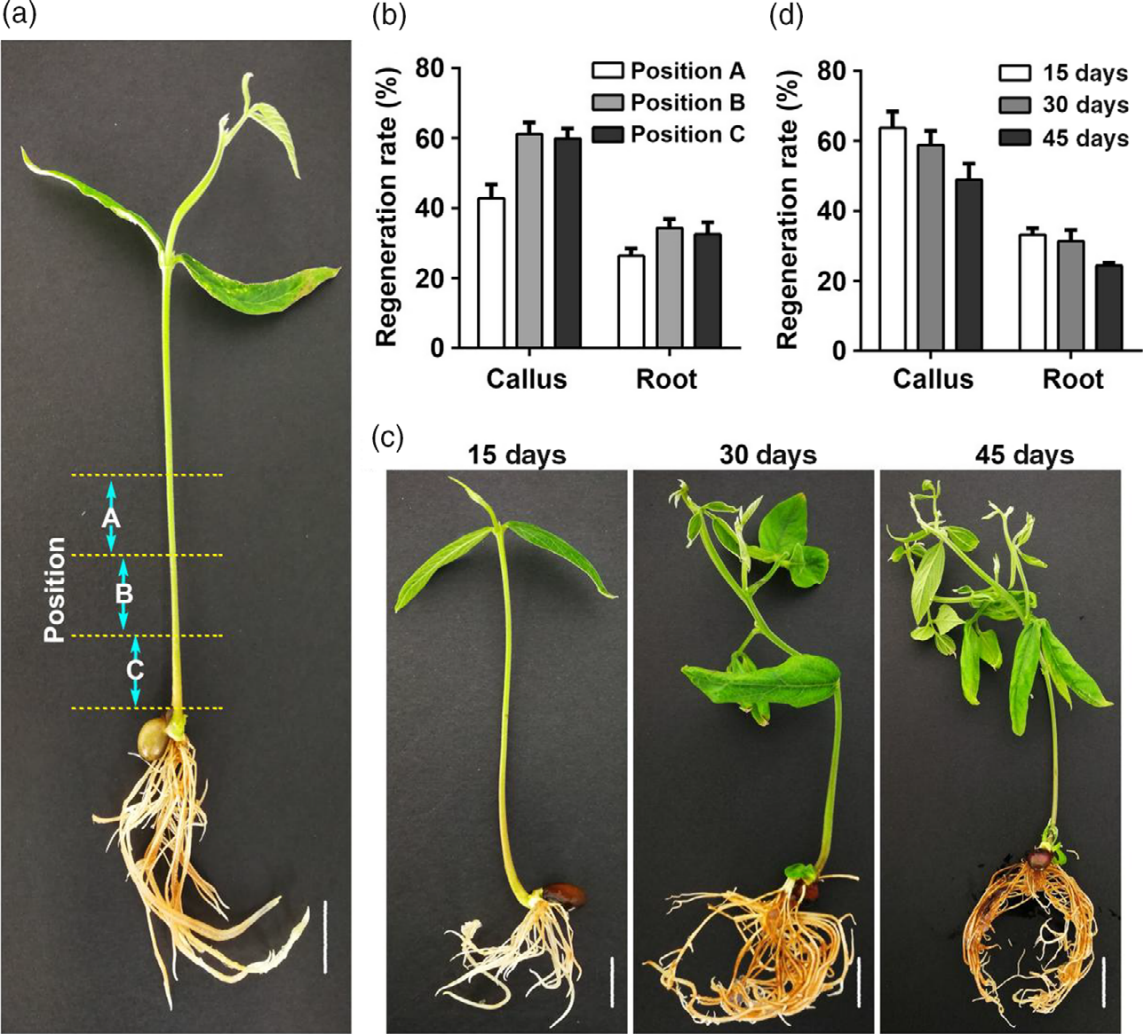
Regeneration rate as affected by injection position and seedling age. (a) fifteen‐day‐old seeding of pigeon pea. From bottom of the stem, three positions are chosen for injection, named position C to A with each position width being about 0.5 cm. (b) Regeneration rate of callus and hairy roots as affected by injection position. (c) Phenotypes of 15‐day‐, 30‐day‐ and 45‐day‐old seedlings. (d) Regeneration rate of callus and hairy roots as affected by the age of seedlings. Bars in A and C is 0.5 cm.

We also tested the optimal injection site and age of seedlings for injection (Figure 3). First, the stem region close to the hypocotyl was divided into three parts with each being 0.5 cm; these were named position A, B, and C respectively (Figure 3a). The induction rate of both calli and hairy roots was around 60% for position B and C whereas it was significantly lower for A (Figure 3b). Next, seedlings at three different ages (15‐day, 30‐day or 45‐day‐old) were used to determine the optimal age of seedlings for injection (Figure 3c). The results show that 15‐day and 30‐day‐old seedlings have higher induction rates than 45‐day‐old seedlings (Figure 3d).

### The applicability of the hairy root transgenic method to other economically important plants

**Figure 4:**
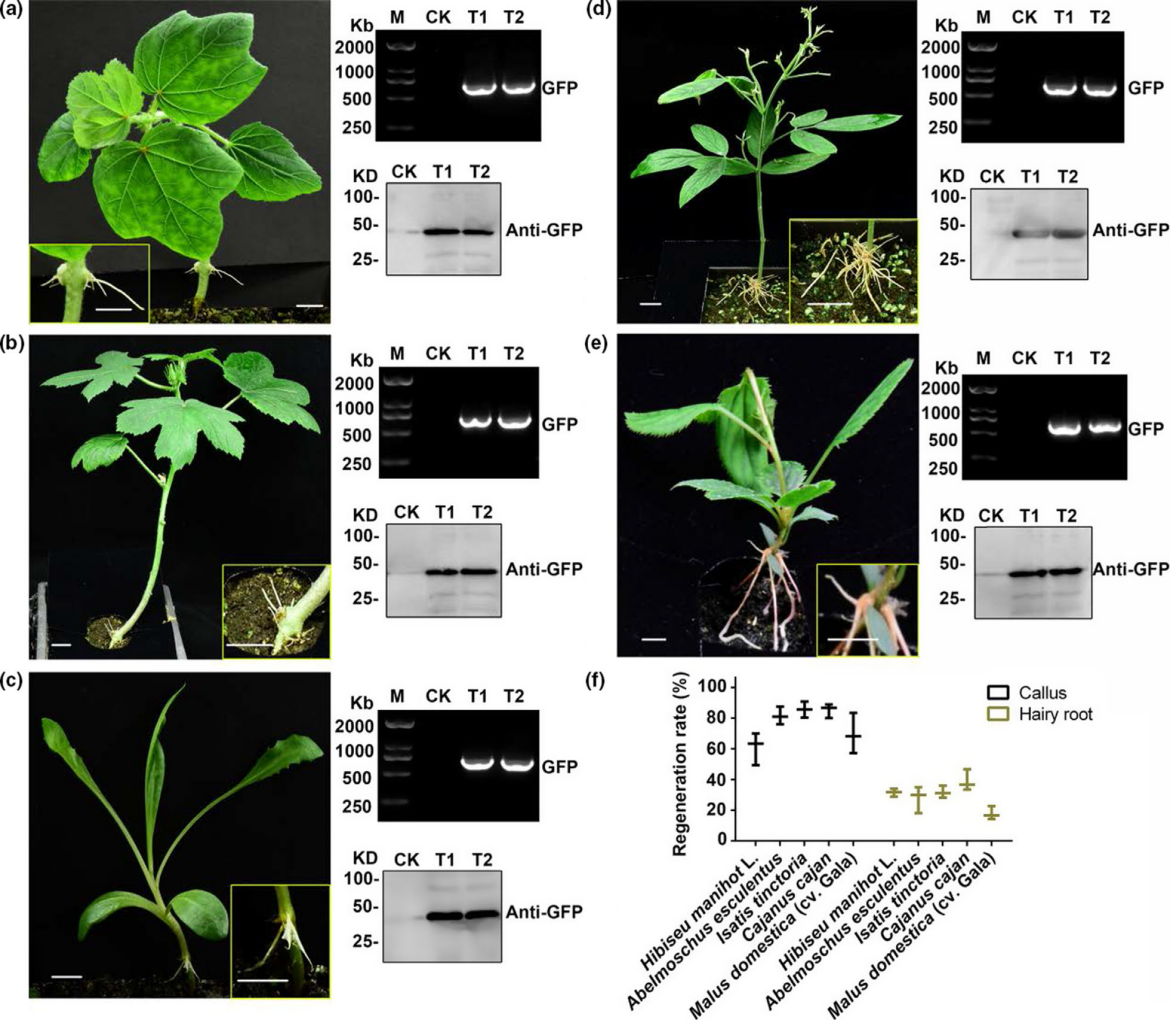
Regeneration of transgenic hairy roots in five typical economically important plants. (a–e) Five plant species with the highest regeneration rates of transgenic hairy roots selected from 12 economically important plants. The pictures showing the hairy root phenotype of *Hibiseu manihot* L, *Abelmoschus esculentus*,* Isatis tinctoria*,* Cajanus cajan* and *Malus domestica* (cv. Gala). On the right side of each picture are RT‐PCR and western blot analyses of each transgenic hairy root lines (T1 and T2 indicate two transgenic lines). Bars, 1 cm. (f) Regeneration rates of callus and hairy roots in the five plant species.

To extend this method to other economically important plants, especially trees and medicinal plants, we selected 12 plant species, including *Hibiseu manihot L, Abelmoschus esculentus, Isatis tinctoria, Cajanus cajan and Malus domestica*, to test the hairy root transgenic system (Table 1, Figure 4). As shown in Table 1, calli were induced on all 12 plants and hairy roots were successfully generated in nine out of the 12 species using this method with slight modifications, except for *Semen cassiae, Ricinus communis* and *Aquilaria sinensis*. The hairy root regeneration rate was between 15–45% and the callus regeneration rate varied between 16–85%. The phenotypes of five selected plant species with the highest root regeneration rates are shown in Figure 4. RT‐PCR and western blot confirmed that the target gene (GFP) was expressed in transgenic hairy roots. These results indicate that the hairy root method has a broad application range from herbs to woody plants.

**Table 1:** Information for hairy root transgenic method used in different plant species

Type	Name	Seeding age, days	Scenario	Strain	OD Value	Regeneration time for callus (Day)	Regeneration rate for callus (%)	Rooting time (Day)	Transgenic root induction efficiency (%)
Herb	*Carthamus tinctorious*	21	III	K599	0.2–0.3	14–17	52 ± 6	21–35	22 ± 4
Herb	*Semen Cassiae*	21	III	K599	0.2–0.3	7–19	38 ± 13	None	None
Herb	*Isatis tinctoria*	21	III	K599	0.2–0.3	4–10	85 ± 5	14–21	31 ± 5
Herb	*Hibiseu manihot* L	21	III	K599	0.2–0.3	14–17	61 ± 11	26–35	32 ± 4
Herb	*Abelmoschus esculentus*	21	III	K599	0.2–0.3	7–18	85 ± 8	15–25	28 ± 11
Herb	*Ricinus communis*	21	III	K599	0.2–0.3	16–21	16 ± 5	None	None
Woody	*Cajanus cajan*	15	I	K599	0.2–0.3	7–14	85 ± 5	14–21	39 ± 7
Woody	*Caragana sinica (Buchoz) Rehd*	21	I	K599	0.2–0.3	7–18	70 ± 22	14–25	19 ± 15
Woody	*Aquilaria sinensis*	40	III	K599	0.3–0.4	14–18	30 ± 14	None	None
Woody	*Malus domestica* (cv. Gala)	40	II	K599	0.3–0.4	18–32	70 ± 13	38–45	18 ± 4
Woody	*Malus hupehensis*	40	II	K599	0.3–0.4	18–35	70 ± 10	40–48	16 ± 4
Woody	*Malus pallasiana*	40	II	K599	0.3–0.4	18–40	68 ± 2	38–50	15 ± 4

### Effects of seedling culture scenarios on hairy root induction rate

**Figure 5:**
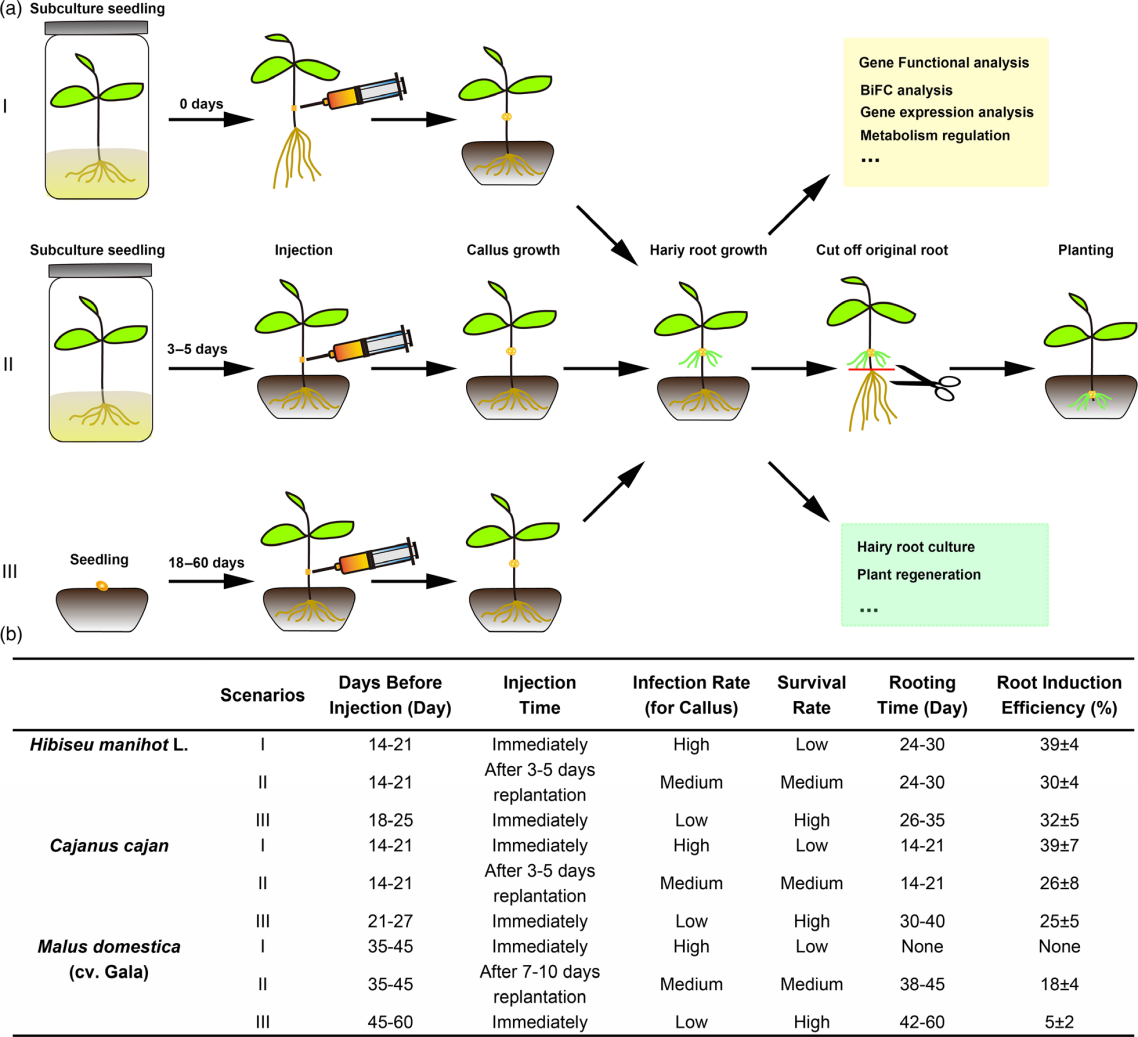
Different scenarios for the hairy root transgenic method used for herbs, shrubs and trees. (a) Flow chart of the hairy root transgenic method. Scenario I, seedlings were cultured in the MS medium first and then picked up from the culture bottle for injection. Scenario II, sub‐cultured seedlings were planted in the soil medium after transfer from culture bottle for about 3–5 days before injection. Scenario III, seedlings were planted in the soil directly before injection. The colour text box showing the possible scope of application of the hairy root transgenic system. (b) Three typical plants, *Hibiseu manihot* L (Herb), *Cajanus cajan* (Shrub) and *Malus domestica* (cv. Gala) (Tree) were selected for the three different scenarios. The injection rate, rooting time and hairy root regeneration rate were listed in the table.

To improve hairy root induction efficiency, we tested three seedling culture scenarios on hairy root induction in three plant species, *H. manihot* L, *C. cajan* and *M. domestica*, representing herbs, shrubs and trees respectively (Figure 5). The three scenarios are: I, after 2–5 weeks of subculture, seedlings were directly injected and then planted into soil medium; II, after 2–5 weeks of subculture, seedlings were planted into soil medium first and then the injection was made after 3–5 days of growth; and III, seedlings were directly planted into soil medium, then injected after 3–8 weeks (Figure 5a). The results show that the time taken for hairy root induction was shorter and hairy root induction rate was higher in scenario I for *H. manihot* L and *C. cajan*, than the other two scenarios. However, for *M.  domestica* (cv. Gala), the survival rate in scenario I was low. When using scenario III, hairy root induction rate was lower than scenario II, but the plants had a higher survival rate (Figure 5b). This indicated that scenario III was more suitable for *M. domestica* (cv. Gala). If we needed an easier and faster method for *H. manihot* L and *C. cajan*, scenario III was best.

### Gene functional analysis using the hairy root transgenic system

**Figure 6:**
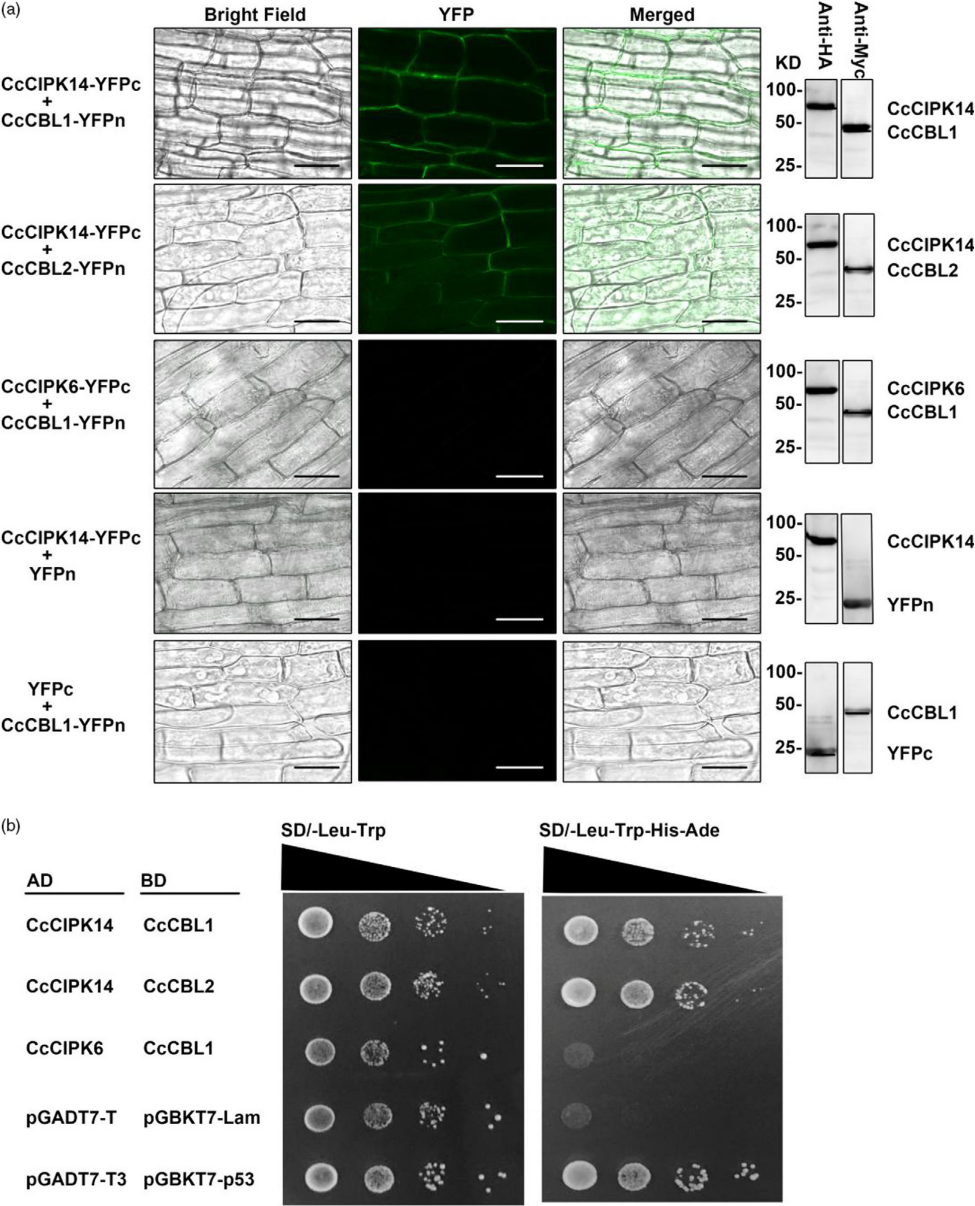
Bimolecular fluorescence complementation (BiFC) assay using the hairy root transgenic system. (a) *CcCIPK14* and *CcCBL1/CcCBL2* were introduced into pSPYNE or pSPYCE vectors respectively. The pairs of CcCIPK6 and CcCBL1, CcCIPK14‐YFPc and YFPn, YFPc and CcCBL1‐YFPn were used as negative controls. The Western Blot on the right side obtained using Myc or HA antibody for the tag at N‐ or C‐terminal YFP respectively, showing the various fusion proteins. Bars, 50 μm. (b) The yeast two‐hybrid assay confirms the binding results of BiFC. CcCIPK14 and CcCBL1/CcCBL2 were introduced into the pGADT7 or pGBKT7 vectors respectively. The pairs of CcCIPK6 and CBL1, pGADT7‐T and pGBKT7‐Lam were used as negative controls. The pair of pGADT7‐p53 and pGBKT7‐p53 was used as positive control. Each colony was dissolved in 100 μL sterile water and then diluted to 10^−1^ to 10^−3^. At least three colonies per combination were tested.

To further confirm that the hairy root system developed here for a wide range of plant species is suitable for conducting functional analysis of genes, bimolecular fluorescence complementation analysis was performed in the hairy root system. The kinase activity of CIPKs (CBL‐interacting protein kinases) modulated by CBLs through direct interaction represents a good example for testing this system. *CcCIPK1*,* CcCIPK14*,* CcCBL1* and *CcCBL2* genes were cloned from the pigeon pea root. Their coding sequences were cloned into pSPYNE and pSPYCE vectors to generate either N‐terminal or C‐terminal YFP (yellow fluorescent protein) fusion proteins respectively (Walter *et al*., 2004). Next, the vectors were transferred into *A. rhizogenes* and injected into the seedling stems. After about 1‐month of culturing transgenic hairy roots, the fluorescent signal was detected using a fluorescence microscope. The results indicated binding of CIPK14 with CBL1 and CBL2 respectively. The negative controls were CIPK6 + CBL1, CIPK14 + YFPn and YFPc+CBL1, which showed no fluorescence signal (Figure 6a). Furthermore, detection of HA or Myc tag by immunoblotting indicated successful expression of the proteins in all hairy root lines. To confirm the BiFC results in the hairy root system, a yeast two‐hybrid assay was performed for CIPK14 and CBL1/CBL2. In Figure 6b, CIPK14 interacted with both CBL1 and CBL2, consistent with the results obtained in the BiFC assay.

### Analysis of the regulation of anthocyanin metabolism in apple

**Figure 7:**
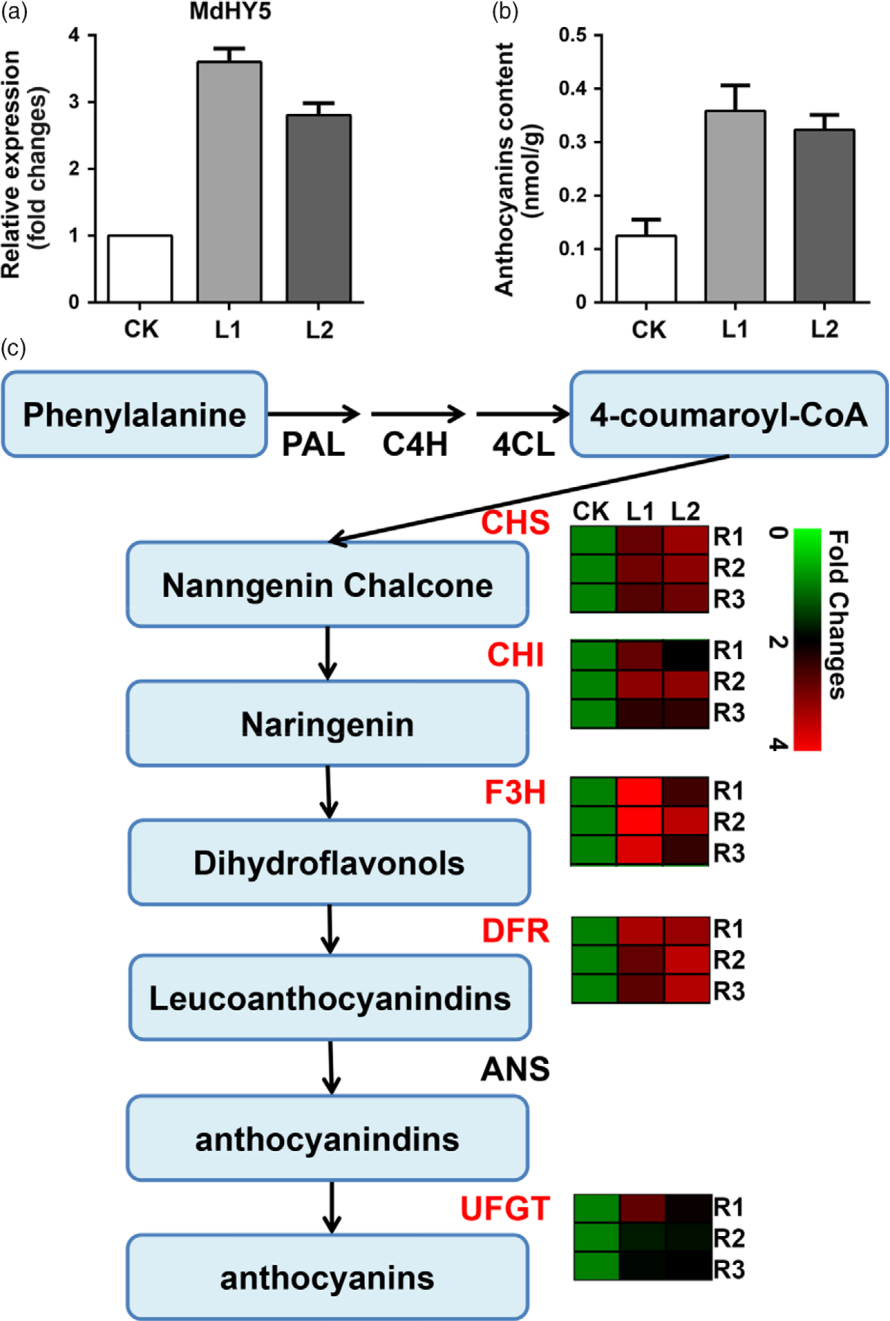
Molecular regulation of anthocyanin synthesis using the hairy root method. (a) Overexpression of MdHY5, a bZIP transcriptional factor, in apple using the hairy root method. The relative expression of MdHY5 in CK (empty vector control) and two transgenic lines, L1 (MdHY5‐OE line 1) and L2 (MdHY5‐OE line 2). (b) Anthocyanin content in both CK and the transgenic hairy roots. (c) The anthocyanin biosynthesis pathway. The expression levels of key enzyme genes, *MdCHS, MdCHI, MdF3H, MdDFR and MdUFGT*, analysed using RT‐qPCR were shown in hotspot map.

The hairy root transgenic method can be also used for functional analysis of genes involved in secondary metabolism. Previous work has shown that MdHY5, a bZIP transcriptional factor, regulates anthocyanin synthesis in apple (An *et al*., 2017). We cloned this gene and overexpressed it in ‘Gala’ using our root transgenic method. The expression level of MdHY5 was enhanced 2.5 to 3‐fold in two transgenic lines, L1 and L2 (Figure 7a). The anthocyanin content in these two lines were increased as expected (Figure 7b). To confirm the role of MdHY5, the expression levels of its target genes in the anthocyanin pathway, including *MdCHS, MdCHI, MdF3H, MdDFR, MdUFGT*, were also detected using RT‐qPCR (Figure 7c). The expression of all these genes were up‐regulated as demonstrated previous (An *et al*., 2017). These results indicate that the hairy root transgenic system can be successfully used to study plant secondary metabolism and offers a simple and fast method to verify the function of genes in both herbs and trees.

## Discussion

Hairy root culture (HRC) technology has been used in hundreds of plants, for example for production of metabolites with recombinant proteins (Abraham and Thomas, 2017; Jiao *et al*., 2018; Kim *et al*., 2002). However, hairy roots for HRC are typically induced from wounded leaves or buds and sub‐cultured in liquid medium to produce molecules or proteins (Abraham and Thomas, 2017; Kim *et al*., 2002). Because HRCs are *in vitro* growth systems, they have major limitations on functional studies of genes *in vivo*, especially those involved in plant development, responses to environmental stresses and secondary metabolism. Research on non‐model plants is often hampered by lack of efficient transformation procedures and/or genomic, proteomic and metabolomic databases, which are still largely limited to model plants, such as *Arabidopsis,* tobacco, tomato and rice to date (Bandaranayake and Yoder, 2018; Hou *et al*., 2017; Matsunami *et al*., 2018). For these non‐model plants, most predicted genes are based on homology analysis and can only be functionally analysed in a heterogenic plant transfer system. In this study, we established a simple and efficient hairy root transgenic system, which allows for stable transformation of roots in a relatively short period of time. More importantly, some woody plants, such as pigeon pea and apple, which currently do not have a highly efficient stable transgenic system or are limited by long growth cycle can also use this method. For example, it typically takes about one year to grow a transgenic apple plant from the initial agrobacterium‐mediated transformation and the efficiency of regeneration of transgenic shoots was low (only 4.6%) in apple cultivar Royal Gala (James *et al*., 1989; Maheswaran *et al*., 1992; Yao *et al*., 1995). Most transformation methods are complex and require tedious work and considerable experience to make them work (Klaas and Jan, 1996; Roberta *et al*., 2017). In the transformation system described here, we can obtain transgenic hairy roots in living plants for testing gene function in 2–3 months.

As a tool for molecular biology research, only a few studies on plants such as soybeans have been reported using the hairy root system to study gene function and root biology (Kereszt *et al*., 2007). In most non‐model plants, researchers have to use transient expression methods or heterologous expression in a model plant for gene function analysis. In either case, it is difficult to screen many candidate genes or accurately verify the original function of the genes. This has led to the study of difficult to transform, yet economically important plants lagging behind the model plants. In this study, we developed a transgenic root method in pigeon pea and extended it to other eleven selected plant species, including woody plants, medicinal plants and fruit trees. Most of them exhibited high efficiency of root regeneration (Table 1, Figure 4). In this system, we used a non‐tissue culture method to induce hairy root in an indoor environment after injection, which also simplified the experimental operation and reduced the difficulty of performing various operations in the tissue culture system (Figure 5). It offers the possibility of screening a large number of candidate genes more quickly and efficiently.

In this work, we first established the root transgenic system for pigeon pea, a woody plant, with a transgenic root induction efficiency of about 39% (Table 1), the transformation rate between positive transformation hairy root and total induced hairy root of about 80% (Figure 2c,i) and a time duration of two months for obtaining transgenic roots (Figure 1, Table 1). We then extended the method to other plants such as *Malus hupehensis*,* Malus pallasiana and I.  tinctoria*. We successfully obtained transgenic roots on most of them at a transformation efficiency between 15% and 31% (Table 1). These results indicate that this system has utility over a wide range of plant species. However, *A. sinensis*,* S. cassiae* and *R. communis* only developed calli, but no transgenic roots. This suggests that either a compatible *A. rhizogenes* strain is required to make the transgenic root method work on a given plant species or the plant status has to be suitable for a given *A. rhizogenes* to infect. So there is a need to screen for more suitable conditions for those plants species that did not work well in this study (Estrada‐Navarrete *et al*., 2006) or to test more *A. rhizogenes* strains on these plants.

We demonstrated that the transgenic root system described here has applications in both plant molecular biology research in identifying and functional characterization of genes and plant genetic engineering/biotechnology. In molecular biology research, we used the most conventional bimolecular fluorescence complementation experiment as an example to demonstrate that the system can be used to verify the interaction between proteins (Figure 6). Compared with the traditional transient expression system, this stable expression system is more reliable and can be used to verify the downstream genes. In genetic engineering research, we chose a bZIP transcriptional factor, MdHY5, with known functions to prove that the method can be used to analyse the molecular regulation of secondary metabolism (Figure 5). The development of this transgenic root system allows for routine molecular approaches such as overexpression and silencing techniques or gene editing tools such as CRISPR/Cas9 (Bortesia and Fischer, 2015; Cai *et al*., 2015) to be applied to non‐model, but economically important plants that currently do not have a transgenic system or even a tissue culture system.

Since the transgenic roots obtained using the method described here can sustain the growth of the whole plant after the original roots are cut off, it forms a unique system with the roots being transgenic but the above‐ground non‐transgenic. This may make it possible to study not only the genes involved in root development and function but also the metabolism of the whole plant in response to gene manipulations in the roots and root to shoot communication in terms of plant hormones, mRNAs and proteins and effects of biotic and abiotic stresses in the soil and rhizosphere.

## Materials and methods

### Plant and bacteria materials

Seeds of pigeon pea [*C. cajan* (L.) Millsp.] were obtained from the Northeast Forestry University in China. Seeds of *Carthamus tinctorious, S. cassiae, I.  tinctoria, H. manihot L, A. esculentus, R. communis, C. cajan, Caragana sinica (Buchoz) Rehd, A. sinensis, M. hupehensis, M. pallasiana* and sub‐cultured *M. domestica* (cv. Gala) were obtained from in our laboratory. The seeds were surface‐sterilized with 0.1% mercuric chloride for 5–10 min and then washed with sterile water five times. Subsequently, the seeds were cultured in the MS medium for about 1–2 months before use. For pigeon pea, the sub‐cultured plants could be injected after about 30 days of growth. For other plant species, plants were transplanted into the soil in 10 cm (diameter) × 9 cm (height) pots containing soil and sand in a 3:1 volumetric ratio and grown in a high humidity environment at 25 °C under fluorescent lights at ~50 μmol photons per m^2^ per s in a 16‐h photoperiod before initiating the injection experiment. Specific seedling age and injection time are provided in Table 1.

*Agrobacterium rhizogenes* strains K599, MSU440, C58C1 and ArA4 were preserved in our laboratory. They were sub‐cultured on YEP medium plates and stored in YEP liquid medium with 15% glycerol at −80°C.

### Hairy root transgenic system

Healthy and uniform sub‐cultured seedling plants were selected for agro‐infiltration using the following injection procedure. Control colonies of *A. rhizogenes* strain K599, MSU440, C58C1 or ArA4 harbouring empty vectors pROK2 or pROK2‐GFP were cultured in 5 mL YEP liquid medium plus 20 mg/L rifampicin and 50 mg/L kanamycin with shaking (180 rpm) at 28°C for about 12–14 h. After the OD_600_ value reached to 0.2–0.6 (Table 1), the cultures were centrifuged at 4722 *g* for 10 min at room temperature and then re‐suspended in MES buffer (10 mm MES‐KOH, pH 5.2, 10 mm MgCl_2_ and 100 μm acetosyringone). As shown in the flow chart of Figure 1, 0.1 mL of Agrobacterium suspension was injected into attached plant stem position C (Figure 3). Ten days after injection (see Table 1 for specific time for each plant species), calli grew on the injection site of the stem. After about one month, when the hairy roots developed well, the original roots were cut off and the transgenic rate for the hairy roots was detected using PCR. The efficiency was calculated as: Transgenic root induction efficiency = (Positive transgenic hairy root plants/total numbers of injected plants)*100%; Transformation rate (pigeon pea) = (Positive transgenic hairy root plants/total induced hairy root plants) *100%.

### RNA, DNA isolation and PCR analysis

Root RNA was extracted using the CTAB method (Meng *et al*., 2018). The quantification of RNA was done using a NanoDrop spectrophotometer (NanoDrop Technologies, Inc, Wilmington, DE). After treatment with RNase‐free DNase I, 1 μg total RNA was reverse‐transcribed to cDNA using the Invitrogen^™^ SuperScript^™^ (Invitrogen, Carlsbad, CA). The primers used for RT‐PCR are listed in the Table S2.

### Bimolecular Fluorescence Complementation (BiFC) assay in hairy root system

Coding sequences of *CcCBL1, CcCBL2* and *CcCIPK6, CcCIPK14* were cloned into the pSPYNE and pSPYCE vectors to generate either N‐terminal or C‐terminal YFP (yellow fluorescent protein) fusion proteins respectively. Then the plasmid pairs (CcCIPK6 + CcCBL1, CcCIPK14 + CcCBL1, CcCIPK14 + CcCBL2) were introduced into *A. rhizogenes* K599 and injected into the sub‐cultured plant stem as described above. After about 20 days, the small hairy root grew. It was imaged using a Leica SP8‐X fluorescence microscope (Leica FluoView SP8) and the expression of various fusion proteins was confirmed by using HA or Myc tag antibody using immunoblotting.

### Yeast two‐hybrid (Y2H) assay

The Y2H assay was performed as YeastmakerTM Yeast Transformation User Manual (Clontech, Saint‐Germain‐en‐Laye, France). The pGBKT7 and pGADT7 empty vector pairs were used as negative control whereas the pGBKT7‐p53 and pGADT7‐T pair were used as a positive control. Coding sequences of *CcCBL1, CcCBL2* were cloned into the pGBKT7 vector as a bait and the coding sequences of *CcCIPK1, CcCIPK14* were cloned into the pGADT7 vector as a prey.

The pairs of vectors were co‐transformed into the yeast strain AH109 and grown on SD/‐Leu‐Trp medium respectively. After culture for 3–6 days, the clones were transferred into the SD/‐Leu‐Trp‐His‐Ade medium at 30°C for 3–4 days. To confirm the results, positive clones were spotted in serial dilutions of yeast (1:1, 1:10, 1:100 and 1:1000) and cultured on the SD/‐Leu‐Trp‐His‐Ade medium.

### Measurement of the total anthocyanin content

Total anthocyaninins in apple were extracted according to the methanol‐HCl method (Lee and Wicker 1991). Half‐gram samples were incubated in 5 mL of 1% (v/v) methanol‐HCl in the dark for about 24 h at room temperature. After that, the absorbance of the extracts was measured using a spectrophotometer (UV‐1600; Shimadzu, Nishiki‐cho, Chiyoda‐ku, Tokyo). The anthocyanin content was calculated as: OD =  (A530‐A620) − 0.1 (OD650‐OD620).

## Author contributions

D.M., Q.Y. and Y.J.F. planned and designed the experiments. D.M., Q.Y., L.L.N., B.Y.D., Z.H.S., L.T.W., H.Y.C. and H.H.L. performed the experiments and analysed the data. D.M. and Q.Y. wrote the manuscript with inputs from all the other authors.

## Conflicts of interest

The authors declare no conflict of interest.

## Supporting information


**Table S1** Regeneration rate of using four *A. rhizogenes* in *Cajanus cajan*.

**Table S2** List of primers used in this study.
