# Supplementary material for: Development of an efficient root transgenic system for pigeon pea and its application to other important economically plants
Source: Plant Biotechnol J. 2019 Mar 27;17(9):1804–13. doi: 10.1111/pbi.13101 (PMC6686128; doi:10.1111/pbi.13101)
Supplement: Supplementary file 1 — Table S1 Regeneration rate of using four A. rhizogenes in Cajanus cajan. [file PBI-17-1804-s002.docx]

| **Table S1 Regeneration rate of using four *A. rhizogenes* in *Cajanus cajan*** | | | | |
| --- | --- | --- | --- | --- |
|  | **MSU440** | **K599** | **C58C1** | **ArA4** |
| **Callus (%)** | 10±2 | 85±5 | 15±5 | 8±3 |
| **Hairy root (%)** | 5±2 | 39±5 | 8±2 | 5±2 |
