# Supplementary material for: Development of an efficient root transgenic system for pigeon pea and its application to other important economically plants
Source: Plant Biotechnol J. 2019 Mar 27;17(9):1804–13. doi: 10.1111/pbi.13101 (PMC6686128; doi:10.1111/pbi.13101)
Supplement: Supplementary file 2 — Table S2 List of primers used in this study. [file PBI-17-1804-s001.docx]

| **Table S2 List of primers used in this study** | | | |
| --- | --- | --- | --- |
| **Primer Name** | **GeneID** | **Forward Primer (5'-3')** | **Reverse Primer (5'-3')** |
| MdCHS | CN944824 | GGAGACAACTGGAGAAGGACTGGAA | CGACATTGATACTGGTGTCTTCA |
| MdCHI | CN946541 | GGGATAACCTCGCGGCCAAA | GCATCCATGCCGGAAGCTACAA |
| MdF3H | CN491664 | TGGAAGCTTGTGAGGACTGGGGT | CTCCTCCGATGGCAAATCAAAGA |
| MdDFR | AF117268 | GATAGGGTTTGAGTTCAAGTA | TCTCCTCAGCAGCCTCAGTTTTCT |
| MdUFGT | AF117267 | CCACCGCCCTTCCAAACACTCT | CACCCTTATGTTACGCGGCATGT |
| MdHY5 | MDP0000586302 | AGAGCAGGCGACGAGCTCCCT | TCTGCTGGATTTCTTCCTCTC |
| MdActin | CN938023 | TGACCGAATGAGCAAGGAAATTACT | TACTCAGCTTTGGCAATCCACATC |
| GFP |  | ATGGTGAGCAAGGGCGAGGAGCTGT | TTACTTGTACAGCTCGTCCAGTGCCG |
| CcCIPK6-CDS | LOC109807670 | ATGAGTCATCCTAAGATTAAGCGCC | CTTTGTTTCTCGCATTTGCATATCA |
| CcCIPK14-CDS | LOC109807907 | ATGAATGGGCAAAAAATTAAGCGCC | CAGGAAACCAATACCAAAATCATAT |
| CcCBL1-CDS | LOC109803538 | ATGGGCTGTTTCAACTCAACGGCAA | TGTAACAATTTCATCGACCTCGGAG |
| CcActin | LOC109798310 | CATGCCATCCTCCGTCTTGACTTAG | GCTCGGCTGTGGTGGTGAATG |
